# Total sleep deprivation selectively impairs motor preparation sub-stages in visual search task: Evidence from lateralized readiness potentials

**DOI:** 10.3389/fnins.2023.989512

**Published:** 2023-02-28

**Authors:** Tao Song, Fangchong Du, Lin Xu, Ziyi Peng, Letong Wang, Cimin Dai, Mengmeng Xu, Ying Zhang, Yongcong Shao, Xiechuan Weng, Shijun Li

**Affiliations:** ^1^School of Psychology, Beijing Sport University, Beijing, China; ^2^Department of Xiangshan Road Outpatient General Clinic, The 8th Medical Center of Chinese PLA General Hospital, Beijing, China; ^3^Department of Neuroscience, Beijing Institute of Basic Medical Sciences, Beijing, China; ^4^Department of Radiology, The First Medical Center of Chinese PLA General Hospital, Beijing, China

**Keywords:** sleep deprivation, lateralized readiness potentials, event-related potentials, electroencephalography, motor preparation, visual search

## Abstract

**Introduction:**

Many studies have provided evidence of a damage effect triggered by total sleep deprivation (TSD). However, it remains unclear whether the motor preparation processing is affected by TSD.

**Methods:**

In the current study, 23 volunteers performed a stimulus-response compatibility visual search task before and after TSD while undergoing spontaneous electroencephalography (EEG).

**Results:**

Repeated-measures analysis of variance revealed that: Compared with that at baseline, the visual search task’s accuracy decreased after TSD, while the response time variance increased significantly. The peak amplitude of the stimulus-locked lateralized readiness potential (LRP) induced by a compatible stimulus was significantly more negative than that induced by an incompatible stimulus before TSD, whereas this difference was not significant after TSD. However, when taking sleep status into consideration, there were no significant main or interaction effects on response-locked LRPs.

**Discussion:**

Our findings suggest that TSD damages visual search behavior, selectively impairs the earlier sub-stages of motor preparation (sensory integration). These findings will provide a new perspective for understanding the effects of sleep loss.

## 1. Introduction

There is increasing evidence that sleep influences the risk of cardiovascular diseases (St-Onge and Zuraikat, 2019). The development of smart devices has altered leisure-time activities and work schedules (Chang et al., 2015), and this has contributed to the growth of the sleep-restricted population. Therefore, exploring how sleep deprivation influences human cognitive functions is critical for preventing its deleterious effects. In numerous studies, shortened sleep is operationalized as total sleep deprivation (TSD), which is a useful metric that comprehensively examines how shortened sleep jeopardizes cognition (Lowe et al., 2017). Previous studies have suggested that TSD impairs different levels of cognitive function; for example, attention performance (measured using vigilant attention and psychomotor vigilance tasks) is significantly weakened by TSD (Lim and Dinges, 2008; McMahon et al., 2018; Gibbings et al., 2020; Stepan et al., 2020). TSD also impairs higher-order cognitive processes such as executive functions (Aidman et al., 2018; Honn and Hinson, 2019), long-term memory (Ratcliff et al., 2018), risky decision-making (Acheson et al., 2007), emotion processing (Kahn et al., 2013; Ben et al., 2020), even social cognitive abilities (Boardman et al., 2017; Deliens and Bukowski, 2018). In addition, studies from animal models have demonstrated that TSD affects various cognitive functions (e.g., Kumar and Jha, 2012; Qureshi and Jha, 2017).

The mechanisms underlying TSD-induced dysfunctions have been explored to a certain degree using cognitive neuroscience technologies. Numerous studies have shown that the reduced activity in the dorsolateral prefrontal and parietal cortexes is correlated with detrimental effects on working memory (Chee and Choo, 2004; Habeck et al., 2004; Choo et al., 2005; Chee and Chuah, 2007; Lythe et al., 2012). Using a stop-signal task with simultaneous electroencephalography (EEG), Kusztor et al. (2019) identified three subcomponents of cognitive control (sustained attention, automatic bottom-up processing, and strategic top-down control) and found that TSD triggered a decline in sustained attention and reduced P3 and P-e amplitudes, suggesting a progressive impairment in top-down control rather than in the other two subcomponents. Trujillo et al. (2009) examined TSD-induced deficits in exogenous and endogenous attention using an adapted attention network test. They demonstrated that TSD affected the early stage, which was indexed through the N1 component, of endogenous attention, whereas the early stage of exogenous attention processes showed a less significant effect (Trujillo et al., 2009). Previous studies had also made an exploration of the relationship between sleep-associated oscillating waves and cognitive function, including both human studies (e.g., Gibbings et al., 2020; Zhang et al., 2023) and animal models (e.g., Tripathi et al., 2018).

While a large amount of research has focused on the neural correlates of the alterations brought about by TSD, there have been almost no investigations of the neural processes involved in motor preparation to date, despite these processes being highly relevant to human reactions. The lateralized readiness potential (LRP) is an event-related potential (ERP) that reflects the activation of the contralateral motor cortex (M1) during voluntary movement (Shibasaki and Hallett, 2006; Smulders and Miller, 2012). A large amount of evidence from physiological studies unambiguously indicates that M1 is the principal generator of LRPs (De Jong et al., 1988; Coles, 1989). LRPs are widely used in many fields of research to assess the processes of motor preparation and execution (Smulders and Miller, 2012; Debnath and Franz, 2016; Jost et al., 2017; Dayan-Riva et al., 2021; Morand-Beaulieu et al., 2021). The LRPs can be divided into two categories: stimulus-locked LRP (s-LRP) reflects sensory integration, while response-locked LRP (r-LRP) is thought to be related to the subsequent processes involved in response execution (Mordkoff and Gianaros, 2000; Rinkenauer et al., 2004; Smulders and Miller, 2012). The sub-stages of motor preparation that are affected by other variables can be accurately identified (e.g., Cheval et al., 2018; Van Voorhis et al., 2019; Qian and Gao, 2021).

Although a large number of studies in the field of cognitive neuroscience of shortened sleep used the ERP technique, there has been almost no research on motor preparation and LRPs. Only Stojanoski et al. (2019) investigated the impact of reduced vigilance using the psychomotor vigilance task following at-home mild sleep restriction. They found that mild sleep restriction reduced the amplitude of LRPs, which indicates that it can negatively affect motor preparation and execution. However, the most serious flaw of their study was that all responses were made with the right hand; in other words, the handedness variable rendered their findings ambiguous. Additionally, the electrodes used to extract the characteristics of LRPs were not as precise as standard paradigms, and the two sub-stages of LRPs were not classified. In this study, we addressed these shortcomings. In the current study, we employed the LRP index to determine whether TSD influences motor preparation processes and to further investigate which sub-stage of motor preparation is influenced by TSD. When responses are made with both hands, the LRPs are easily observed in a stimulus-response compatibility task (Gorman Bozorgpour et al., 2013; Van Voorhis et al., 2019; Nayak et al., 2020; Dayan-Riva et al., 2021; Morand-Beaulieu et al., 2021). Given that a systematic study by Clark et al. (2015) in which various ERP components were measured found that the “visual search task” was suitable for analyzing the two types of LRPs, we employed this method in the current study to analyze these potentials. In the current study, we designed the present study to quantify the LRPs evoked by a stimulus-response compatibility visual search task and explore whether TSD damages the motor preparation process. We hypothesized that: (i) TSD would affect motor preparation function; (ii) TSD impaired two sub-stages of motor preparation (sensory integration and response execution).

## 2. Materials and methods

### 2.1. Participants

Twenty-four young male volunteers (22.91 ± 2.26 years) who were healthy university students in Beijing participated in this study. All were right-handed, had normal or corrected vision, and had no history of neurological or mental disorders. The experimental protocol was approved by the Ethics Committee of Beihang University (approval number BM20180040). All participants maintained healthy sleep habits (Pittsburgh Sleep Quality Index score < 5) (Buysse et al., 1989). The participants were instructed to ensure adequate sleep (7–9 h every day) for half a month before the beginning of the TSD experiment. All participants declared that they did not have a habit of smoking cigarettes, that they did not drink alcohol or coffee, and that they had not taken any medications within 48 h before the experiment. Each participant signed an informed consent form before the beginning of the experiment.

### 2.2. Experimental design

**FIGURE 1:**
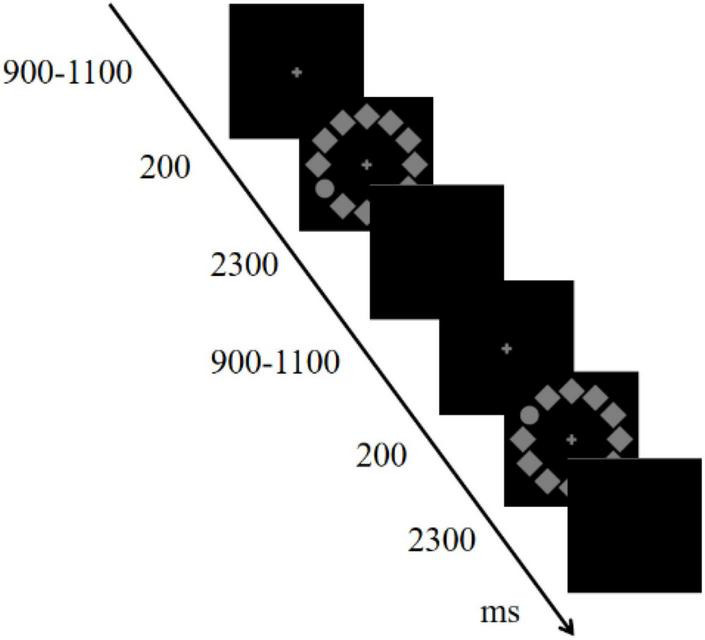
Sequence of the stimulus-response compatibility visual search task.

The stimulus sequence for the visual search task is shown in Figure 1. A circular search array consisted of 12 items (1.7°× 1.7°, 50% contrast, 13.5 cd/m^2^), 11 diamonds, and one circle (target) positioned on a black background. The target was randomly located at either 2/4 o’clock (right) or 8/10 o’clock (left) with equal probability. All subjects were instructed to maintain their gaze at the center of the screen, respond to the position of the target (upper or lower), and ignore other extraneous items. Participants assigned odd numbers pressed the F key with their left hands for the upper stimulus and the J key with their right hands for the lower stimulus; those assigned even numbers followed these rules in reverse. The combination of the position of the target (right or left) and the responding hand produced stimuli that were either compatible (right-right, left-left) or incompatible (left-right, right-left). Each trial started with a fixation cross displayed for 900–1,100 ms randomly, followed by the stimulus array for 200 ms, and a blank screen for 2,300 ms; participants responded during the blank screen interval. The experiment consisted of two 120-trial blocks and lasted approximately 10 min. The design of the visual search task was based on previous studies by Sun et al. (2018) and Wang et al. (2016). All the participants completed the task using the same computer.

### 2.3. Experimental procedures

**FIGURE 2:**
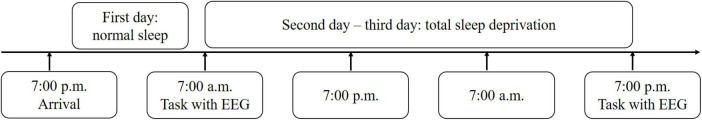
Timelines of the total sleep deprivation (TSD) experiment. EEG, electroencephalography.

The experimental procedure is illustrated in Figure 2. Two participants conducted the experiment simultaneously. The participants arrived at the laboratory the day before the experiment and slept there that night to ensure adequate sleep (i.e., more than 8 h). Before commencing the experiment, all participants performed 20 trial runs of the visual search task to ensure that they had mastered the task’s requirements. The following day, the participants completed the visual search task with simultaneous EEG recordings (baseline readings) at 8:00 am. After these baseline recordings were obtained, the participants stayed in the laboratory for the entire duration of their sleep deprivation; they were offered refreshments and were permitted to play games and watch movies. After 36 h of TSD, they completed the same task while again subject to simultaneous EEG recordings (TSD readings). During each EEG recording, the two participants completed the visual search task separately in randomized order. The participants were prevented from taking any drugs/stimulating agents during the TSD period. At least two paramedics accompanied and observed the participants and reminded them to remain awake throughout the entire experiment.

### 2.4. Behavioral data analysis

Three behavioral performance measures during the visual search task were recorded for analysis under both baseline and TSD conditions. Trials with error responses and those with response times (RT) less than 200 ms or more than 2,000 ms were excluded from the behavioral and subsequent LRP data analyses. The accuracy was calculated as the number of correct trials meeting the RT range divided by the total number of trials. The RT was the average reaction time of all correct trials meeting the RT range. The RT_SD_ was calculated as the variance of RT. One participant’s behavioral performance was negative (accuracy = 14.58%), and his data were therefore excluded from the analysis as well. Descriptive statistics are presented as means ± standard deviations. Repeated-measures analysis of variance (ANOVA) was used to evaluate accuracy and RT. The main effects and interactions between sleep conditions (baseline and TSD) and stimulus types (compatible and incompatible) were also analyzed. The estimates of effect size were reported as partial η^2^ or Cohen’s d, and the statistical power was estimated as power. These analyses were performed using SPSS Statistics for Windows, version 22.0 (IBM Corp., Armonk, NY, USA).

### 2.5. EEG recordings and preprocessing

Continuous EEG was recorded from an elastic cap with 30 electrodes that were mounted according to the 10–20 system standard positions using a SynAmps2 amplifier (Compumedics Neuroscan, Victoria, Australia). The online sampling rate was 1,000 Hz, and the impedance of each electrode was reduced and maintained below 5 kΩ. The vertical eye movements were monitored with two electrodes placed 10 mm above and below the left eye, whereas the horizontal eye movements were monitored with two electrodes placed on the left and right temples. The reference electrodes were placed bilaterally on the mastoids.

Raw EEG data were preprocessed offline using MATLAB R2017a (The MathWorks, Inc., Natick, MA, United States)^1^ that incorporated the EEGLAB2020_0 toolbox^2^ (Delorme and Makeig, 2004). The offline sampling rate was reduced to 250 Hz, and the average reference was used for re-referencing. A band-pass filter of 0.1–30 Hz was used by a 6th order Butterworth filter with a frequency slope of 36 dB/oct *via* the ERPLAB plugin^3^ (Lopez-Calderon and Luck, 2014). After independent component analysis, elements symbolizing eye movement and inordinate muscle activity were identified using two auxiliary plugins for artifact recognition, ICLabel^4^ (Pion-Tonachini et al., 2019) and Adjust^5^ (Mognon et al., 2011), and were removed manually. Epochs with a length of 600 ms ranging from −200 to 400 ms with respect to the onset of the stimuli as well as those with a length of 600 ms ranging from −500 to 100 ms with respect to the onset of the motor response were then extracted from the continuous EEG data to determine the s-LRP and r-LRP, respectively. The s-LRP was baseline-corrected in the range of −200 ms to 0 ms before stimulus onset, while the r-LRP was baseline-corrected in the range of −500 to −300 ms before response onset. The baseline correction was performed by subtracting the mean activity during the corresponding baseline period from the segmented data. The trials with voltages exceeding ± 75 μV in any channel were detected using a sliding time window with a length of 200 ms, and were excluded from the ERP grand average automatically *via* the ERPLAB plugin (see text footnote 3; Lopez-Calderon and Luck, 2014). One participant’s ERPs data were excluded from the analysis because of the tiny ratio of valid to invalid epochs (i.e., less than one). According to Luck (2005), the numbers of included trials under the two sleep conditions were sufficient to produce stable results and draw robust conclusions.

### 2.6. LRPs data analysis

The characteristics of LRPs were extracted using the ERPLAB plugin (see text footnote 3; Lopez-Calderon and Luck, 2014) after averaging and calculating data from only the corrected responses post-screening. The LRPs are calculated as the difference in amplitude between the contralateral and ipsilateral electrodes located near M1 to the responding hand (Slobounov, 2010). The C3 and C4 electrodes were targeted to calculate LRPs using the averaging method LRP = (mean [C4-C3]^left hand^ + mean [C3-C4]^right hand^)/2 (Coles, 1989). We chose the time windows for the two types of LRP in this study based on the definition of LRPs (Mordkoff and Gianaros, 2000; Rinkenauer et al., 2004; Smulders and Miller, 2012) and the waveforms we obtained. The peak amplitude and onset latency of s-LRP were measured from 200 to 350 ms as governed by the onset of the stimuli, while the peak amplitude and onset latency of r-LRP were measured from −200 to 0 ms as governed by the onset of the motor response. A 2 (sleep conditions: baseline, TSD) × 2 (stimulus types: compatible, incompatible) repeated-measures ANOVA was used to analyze the peak amplitudes of the LRP components. The main effects and interaction effects between sleep conditions and stimulus types were analyzed separately. The estimates of effect size are reported as partial η^2^ or Cohen’s d. A jackknife-based method was applied to extract the onset latencies of the LRPs (Miller et al., 1998). Twenty-one subsamples of grand average LRPs were calculated by excluding the LRP data of a different participant under each sleep condition. The onset latencies of the LRPs were defined as 50% of the peak amplitude. Statistical analyses were conducted using the corrected *F*-values approach (*F*_*c*_ = *F*/[n−1]^2^) or corrected *t*-values approach (*t*_*c*_ = *t*/[n−1]), where *F*_*c*_ and *t*_*c*_ denote the corrected values and n denotes the number of participants (Kiesel et al., 2008). These analyses were performed using the SPSS software.

## 3. Results

### 3.1. Behavioral results

The means and standard deviations of the behavioral performance measures (accuracy, RT, and RT_*SD*_) are shown in Table 1.

**TABLE 1:** Means and standard deviations of various measures of behavioral performance and lateralized readiness potentials.

	At baseline		After sleep deprivation	
	Compatible	Incompatible	Compatible	Incompatible
Accuracy	0.97 ± 0.03	0.90 ± 0.08	0.94 ± 0.06	0.84 ± 0.10
RT(ms)	482.09 ± 91.45	537.33 ± 74.21	487.96 ± 77.36	545.26 ± 85.32
RT_SD_ (ms)	106.07 ± 35.02	108.43 ± 31.33	117.1 ± 40.58	135.85 ± 55.60
s-LRP peak amplitude (μV)	–4.58 ± 3.53	–3.98 ± 3.44	–3.23 ± 2.52	–3.42 ± 3.07
s-LRP onset latency (ms)	220.00 ± 1.21	221.45 ± 1.92	226.91 ± 1.78	220.18 ± 0.83
r-LRP peak amplitude (μV)	–3.59 ± 3.27	–1.13 ± 1.91	–2.95 ± 2.29	–1.79 ± 1.46
r-LRP onset latency (ms)	–185.64 ± 4.92	–104.18 ± 5.59	–177.45 ± 3.09	–109.64 ± 2.87

r-LRP, response-locked lateralized readiness potential; RT, response time; RT_SD_, response time variance; s-LRP, stimulus-locked lateralized readiness potential.

#### 3.1.1. Accuracy

**FIGURE 3:**
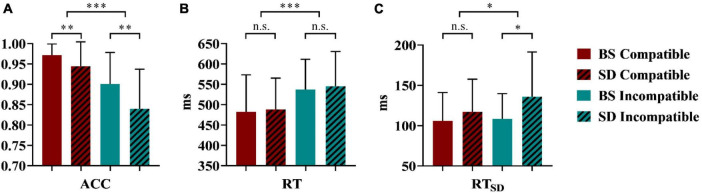
Behavioral results of ACC **(A)**, RT **(B)**, RT_SD_ **(C)** in the stimulus-response compatibility visual search task after a full night’s sleep (at baseline) versus after total sleep deprivation (TSD). ACC, accuracy; RT, response time; RT_SD_, response time variance; BS, at baseline; SD, after total sleep deprivation; *p < 0.05; **p < 0.01; ***p < 0.001; n.s., not significant.

As shown in Figure 3A, repeated-measures ANOVA revealed that the interaction effect between sleep status and stimulus types was significant [*F*_(1,22)_ = 4.37, *p* = 0.048, partial η^2^ = 0.17, power = 0.52], suggesting that the accuracy of different stimulus types differed across sleep conditions. The simple effect analysis showed that the accuracies of both compatible (*t*_22_ = 2.62, *p* = 0.008, Cohen’s d = 0.58, power = 0.90) and incompatible (*t*_22_ = 3.29, *p* = 0.004, Cohen’s d = 0.69, power = 0.99) stimuli were higher at baseline than those after TSD. The main effect of sleep status was significant [*F*_(1,22)_ = 12.06, *p* = 0.002, partial η^2^ = 0.36, power = 0.91], which indicated that TSD affected accuracy, that is, the accuracy was higher at baseline (0.94 ± 0.07) than after TSD (0.89 ± 0.09). The stimulus types also played a significant role [*F*_(1,22)_ = 40.51, *p* < 0.001, partial η^2^ = 0.65, power = 1.00], suggesting that the accuracy of the compatible stimulus (0.96 ± 0.05) was higher than that of the incompatible stimulus (0.87 ± 0.09).

#### 3.1.2.RT

As shown in Figure 3B, repeated-measures ANOVA of RT revealed a significant effect of stimulus types [*F*_(1,22)_ = 85.01, *p* < 0.001, partial η^2^ = 0.79, power = 1.00]; that is, the RT of the compatible stimulus (485.05 ± 82.89 ms) was faster than that of the incompatible stimulus (541.30 ± 78.30 ms). In terms of sleep conditions, the main effect was not significant [*F*_(1,22)_ = 0.22, *p* = 0.642, partial η^2^ = 0.01, power = 0.07]. The interaction effect between sleep status and stimulus types was not significant [*F*_(1,22)_ = 0.02, *p* = 0.886, partial η^2^ = 0, power = 0.05] as well.

#### 3.1.3. RT_SD_

As shown in Figure 3C, repeated-measures ANOVA revealed that sleep status significantly affected RT_*SD*_ [*F*_(1,22)_ = 4.66, *p* = 0.042, partial η^2^ = 0.18, power = 0.54]; participants exhibited more variability after TSD (126.45 ± 48.52 ms) than at baseline (107.25 ± 32.52 ms). The type of stimulus also had a significant effect [*F*_(1,22)_ = 6.74, *p* = 0.017, partial η^2^ = 0.23, power = 0.70], as the RT_SD_ of the incompatible stimulus (122.14 ± 46.21 ms) was greater than that of the compatible stimulus (111.58 ± 37.48 ms). The interaction effect between sleep status and stimulus types did not meet the threshold for significance [*F*_(1,22)_ = 3.64, *p* = 0.070, partial η^2^ = 0.14, power = 0.45].

### 3.2. LRP

The means and standard deviations of LRP characteristics (peak amplitude and onset latency) are shown in Table 1.

The polarity of the s-LRP induced by the incompatible stimulus was the inverse of that induced by the compatible stimulus; moreover, the polarity of the s-LRP induced by the incompatible stimulus the inverse of that of the r-LRP. These results were consistent with the notion that lateralized sensory potentials are one of the sources of LRP when stimuli were presented to the left or the right of the participant’s midline (Smulders and Miller, 2012). This in turn indicated that the s-LRP reflected sensory integration (Mordkoff and Gianaros, 2000; Rinkenauer et al., 2004). To compare the peak amplitudes and onset latency of the s-LRP between the two sleep conditions, we inverted the polarity; that is, we calculated the s-LRP using the contralateral electrodes of the visual field, where incompatible stimuli appear, rather than the responding hands.

#### 3.2.1. s-LRP

**FIGURE 4:**
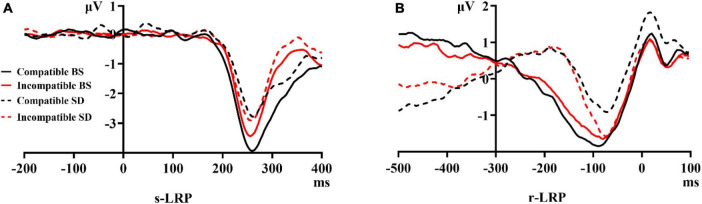
Grand average waves of the s-LRP **(A)** and r-LRP **(B)** induced by the target stimulus of the stimulus-response compatibility visual search task after a full night’s sleep (at baseline) versus after total sleep deprivation (TSD). The polarity of the s-LRP induced by the incompatible stimulus was the inverse of that induced by the compatible stimulus; therefore, we inverted the polarity to compare the peak amplitudes and onset latencies of the s-LRPs between the two sleep conditions. s-LRP, stimulus-locked lateralized readiness potential; r-LRP, response-locked lateralized readiness potential; BS, at baseline; SD, after total sleep deprivation.

The grand-average waves of s-LRP waves were present in Figure 4A.

Repeated-measures ANOVA of peak amplitudes of the s-LRP components revealed that the interaction effect between sleep status and stimulus types was significant [*F*_(1,21)_ = 4.60, *p* = 0.044, partial η^2^ = 0.18, power = 0.54], suggesting that the peak amplitudes of the s-LRP triggered by the two types of stimuli differed across sleep conditions. The simple effect analysis showed that the peak amplitude of the s-LRP induced by a compatible stimulus was significantly more negative than that induced by an incompatible stimulus before TSD (*t_21_* = 2.24, *p* = 0.036, Cohen’s d = 0.48, power = 0.69), whereas this difference was not significant after TSD (*t_21_* = 0.77, *p* = 0.451, Cohen’s d = 0.16, power = 0.11). The main effects of stimulus types [*F*_(1,21)_ = 1.24, *p* = 0.278, partial η^2^ = 0.06, power = 0.19] and sleep status [*F*_(1,21)_ = 2.55, *p* = 0.125, partial η^2^ = 0.11, power = 0.33] were not significant.

Repeated-measures ANOVA of onset latency in the s-LRP components found no significant main effects of sleep status [*Fc*_(1,21)_ = 0.18, *p* = 0.673], stimulus types [*Fc*_(1,21)_ = 0.21, *p* = 0.653], or the interaction effect between them [*Fc*_(1,21)_ = 0.40, *p* = 0.534].

#### 3.2.2. r-LRP

The grand-average waves of r-LRP were presented in Figure 4B.

Repeated-measures ANOVA of peak amplitudes of the r-LRP components revealed that the main effects of stimulus types [*F*_(1,21)_ = 7.87, *p* = 0.011, partial η^2^ = 0.27, power = 0.76] was significant, suggesting that the peak amplitudes of the r-LRP components triggered by a compatible stimulus (−3.27 ± 2.84 μV) was more negative than that triggered by an incompatible stimulus (−1.46 ± 1.73 μV). Otherwise, there was no significant effect of sleep status [*F*_(1,21)_ < 0.01, *p* = 0.966, partial η^2^ = 0, power = 0.05] or interaction between sleep status and stimulus types [*F*_(1,21)_ = 1.66, *p* = 0.212, partial η^2^ = 0.07, power = 0.23].

Repeated-measures ANOVA of the onset latency of the r-LRP components revealed that the main effects of stimulus types [*Fc*_(1,21)_ = 14.48, *p* = 0.001] was significant, suggesting that the onset latency of the r-LRP induced by a compatible stimulus (−181.55 ± 5.8 ms) was earlier than that induced by an incompatible stimulus (−106.91 ± 5.21 ms) when combining the two sleep conditions. The main effects of sleep status [*Fc*_(1,21)_ = 0.01, *p* = 0.939] and the interaction effect between sleep status and stimulus types [*Fc*_(1,21)_ = 0.13, *p* = 0.719] were not significant.

## 4. Discussion

We applied a visual search task that included stimulus-response compatibility to observe the processing of motor preparation by LRPs. Our most important finding was the selective impairments on the two types of LRP, which fully supported hypothesis (i) and partially supported hypothesis (ii). The s-LRP, which is related to the earlier sub-stage of motor preparation (sensory integration), was sensitive to sleep deprivation, while the r-LRP, which is related to the later sub-stage of motor preparation (response execution), was not.

In the current study, TSD decreased the accuracy of the visual search task while increasing the RT variance; this suggested that participants manipulated the visual search task less accurately and with less stability after TSD. These behavioral findings are consistent with those of previous studies, many of which have shown that TSD weakens attention performance (Lim and Dinges, 2008; McMahon et al., 2018; Gibbings et al., 2020; Stepan et al., 2020) and impairs higher-order cognitive processes that are based on attention (such as long-term memory) (Ratcliff et al., 2018). Many studies have suggested that executive function is impaired by TSD (Aidman et al., 2018; Honn and Hinson, 2019). It is more difficult for participants to respond to incompatible stimuli than to compatible stimuli; the cognitive processing of incompatible stimuli include additional cognitive functions such as conflict monitoring and inhibitory control, which are important for central executive functions.

The amplitudes of the LRPs reflect the intensity of neural activity in the M1 area. In terms of the peak amplitudes of the s-LRP components, the significant interaction effect between sleep status and stimulus types indicated that TSD damages the processing of sensory integration. Before TSD, the s-LRP induced by a compatible stimulus was more negative than that induced by an incompatible stimulus. The incompatible stimulus was more complicated than the compatible stimulus. For incompatible stimulus, the sensory integration processes included additional cognitive functions such as conflict monitoring and inhibitory control, which required both contralateral and ipsilateral brain activity. This was not the case with a compatible stimulus. When extracting the amplitude of s-LRP using a subtractive formula, the difference between s-LRPs induced by the two types of stimuli before TSD was pronounced. After TSD, however, the attention was thoroughly weakened to the extent that this difference disappeared. For peak r-LRP component amplitudes, the lack of a significant effect of sleep status as well as the interaction effect between sleep status and stimulus types suggested that TSD seldom affected the later sub-stage of motor preparation (response execution).

The onset latency of LRPs reflects the relative onset time of neural activity in the M1 area. There was no significant difference between the onset latency of s-LRP evoked by two types of stimuli at baseline or after TSD, suggesting that there were no obvious TSD-induced setbacks that would delay the relative onset time of the earlier sub-stage of motor preparation. In the later sub-stage of motor preparation, the onset latency of r-LRP induced by an incompatible stimulus was slower than that induced by a compatible stimulus when combining the two sleep conditions, which suggested that the relative onset time of the response execution is delayed during more complicated cognition processing. This delay is consistent with the postulate that the processing of incompatible stimuli included additional cognitive functions; extra processing such as conflict monitoring between sensory integration and response execution deferred the onset time of the response execution.

An additional observation in the current study was that the polarity of the s-LRP induced by the incompatible stimulus was opposite to that induced by the compatible stimulus. The different polarities of s-LRP and r-LRP evoked by incompatible stimuli provided new evidence supporting the two-stage classification of LRPs (Mordkoff and Gianaros, 2000; Rinkenauer et al., 2004). That is, the difference between the two subcomponents of LRP is not only reflected in the onset latency, but also in the relationship between the position of the stimulus presentation and the brain regions. The contralateral brain hemisphere of the visual field and that of the response hand are the same for compatible stimuli but different for incompatible stimuli. The formula “LRP = (mean [C4-C3]^left hand^ + mean [C3-C4]^right hand^)/2” involved response hands rather than visual fields; therefore, the polarity of the s-LRP induced by the incompatible stimulus was opposite to that induced by compatible stimulus. Hence, when we calculated the s-LRP using the contralateral electrodes of the visual field, the waves of the s-LRP induced by the incompatible stimulus were similar to those induced by compatible stimulus (Figure 4A). The corresponding relationship between the dominant brain hemisphere and the visual fields rather than the responding hands showed that the s-LRP was an underlying mechanism of early sensory integration. Taken together, our data show that stimuli are first processed in the contralateral hemisphere corresponding to the visual field during the former sub-stage of motor preparation (sensory integration), then in the contralateral hemisphere corresponding to the response hand during the later sub-stage of motor preparation (response execution). The interesting question of whether the polarity of the s-LRP is inverted ought to be investigated in future studies.

As mentioned in the section “1. Introduction”, LRPs are widely used in many fields of research to assess the processes of motor preparation. The s-LRP reflects sensory integration, while the r-LRP is thought to be related to the subsequent processes involved in response execution. This study contributed to our knowledge regarding how sleep loss damages motor preparation. Previous study found that mild sleep restriction reduced the amplitude of LRPs in the psychomotor vigilance task, indicating that sleep loss can negatively affect motor preparation processes (Stojanoski et al., 2019). Our study revealed that TSD impaired sensory integration (the earlier sub-stage of motor preparation) rather than response execution (the later sub-stage of motor preparation). In other words, sensory integration (s-LRP) is more susceptible to TSD than response execution (r-LRP).

There were some limitations that deserve consideration in our current study. First, the sample size was not very large, which influenced the generalizability of the findings. Second, other sample-specific features limited the ecological validity; for example, all the participants were young adult males, and future studies ought to broaden characteristics such as sex and age. Third, the number of electrodes used was insufficient; high-density EEG would be more effective for acquiring the signal of M1 activation. Finally, given that the M1 activation in the time window between s-LRP and r-LRP involves a combination of ERP components (e.g., N2 and P3), we did not trace the intermediate processes of s-LRP and r-LRP. More exploratory analyses may be necessary to clarify these intermediate processes in future studies.

In summary, our study revealed that TSD selectively attenuates motor preparation processing; that is, TSD impairs the earlier sensory integration sub-stage rather than the later response execution. These findings provide new evidence regarding the weakened cognitive functions induced by sleep loss.

## Data availability statement

The raw data supporting the conclusions of this article will be made available by the authors, without undue reservation.

## Ethics statement

The studies involving human participants were reviewed and approved by the Ethics Committee of Beihang University. The patients/participants provided their written informed consent to participate in this study.

## Author contributions

YS, XW, and SL designed the experiment. LX, ZP, LW, CD, MX, and YZ collected the experiment data. TS and FD analyzed the data, interpreted the results, and wrote the manuscript. All authors listed have read and approved the manuscript.

## Footnotes

1
https://www.mathworks.com/products/matlab.html

2
https://eeglab.org/

3
https://github.com/lucklab/erplab

4
https://github.com/sccn/ICLabel

5
https://www.nitrc.org/projects/adjust
